# Correction: Filling gaps in bacterial amino acid biosynthesis pathways with high-throughput genetics

**DOI:** 10.1371/journal.pgen.1008106

**Published:** 2019-04-03

**Authors:** Morgan N. Price, Grant M. Zane, Jennifer V. Kuehl, Ryan A. Melnyk, Judy D. Wall, Adam M. Deutschbauer, Adam P. Arkin

The reference list for S1 Text is shown at the end of the S3 Text file. Please view the correct S1 Text and S3 Text files below, which include the correct reference lists for each file.

## Supporting information

S1 Text Testing the auxotroph predictions of D’Souza and colleagues. (PDF)

S3 Text Clear candidates whose mutants were not consistently auxotrophic. (PDF)
