# Supplementary material for: Filling gaps in bacterial amino acid biosynthesis pathways with high-throughput genetics
Source: PLoS Genet. 2018 Jan 11;14(1):e1007147. doi: 10.1371/journal.pgen.1007147 (PMC5764234; doi:10.1371/journal.pgen.1007147)
Supplement: S1 Text — (PDF) [file pgen.1007147.s003.pdf]

S1 Text: Testing the auxotroph predictions of D'Souza and colleagues

D'Souza and colleagues [7] predicted that most bacteria cannot make all 20 amino acids. For example, they predicted that over 30% of sequenced bacterial genomes do not encode the capability to synthesize phenylalanine. Dr. Christian Kost kindly provided us with the list of predictions for phenylalanine auxotrophy (personal communication, September 16, 2014) and we tested these predictions in two ways. First, of the 10 bacteria we focused on here, they had predictions for five. Two of these five bacteria were predicted to be auxotrophic for phenylalanine (*S. meliloti* 1021 and *D. vulgaris* Miyazaki F). We believe that these predictions are based on the genome sequences of the exact same strains that we studied. This suggests that many of their auxotroph predictions are false positives, but five bacteria is a small sample. To test the predictions on a larger sample of bacteria, we considered nitrogen-fixing bacteria. These bacteria should be particularly unlikely to be auxotrophic for amino acids, as amino acids provide a more biochemically convenient source of nitrogen than nitrogen gas. Also, nitrogen fixation is usually studied by growing bacteria in the absence of amino acids. So, we compared a list of known nitrogen-fixing bacteria with sequenced genomes [53] to the predictions provided by Dr. Kost. We were careful to make sure that the strain identifiers matched. We excluded cyanobacterium UCYN-A from consideration because it is an endosymbiont [54]. This left us with predictions for 39 nitrogen-fixing strains. 11 of the 39 strains (28%) are predicted to be auxotrophic for phenylalanine, which is about the same rate as for other bacteria (36%). The difference between the two proportions is not significantly different ($P = 0.39$, Fisher exact test). We then examined a random sample of five of the 11 "auxotrophic" nitrogen-fixing bacteria to check that they grow in minimal media. One of these bacteria was *S. meliloti* 1021, which grows in minimal media as long as cobalt and biotin are provided [12]. For the other four, we found published reports of growth in minimal media with no amino acids. In general, these media contain inorganic salts, buffer, trace vitamins, and a carbon and/or nitrogen source, and none of the media contain amino acids. Specifically, *Clostridium beijerinckii* NCIMB 8052 grows in MP2 medium with acetate as carbon source [55]. *Clostridium kluyveri* DSM 555 is also known as ATCC 8527 and grows in Stadtman-Burton medium with ammonia or N_2 as the sole source of nitrogen [56]. *Methylocella silvestris* BL2 grows in NMS media with methane as the sole source of fixed carbon [57]. And the cyanobacterium *Trichodesmium erythraeum* IMS101 grows in YBC-II medium with CO_2 and N_2 as the major nutrients [58]. Overall, we found that the D'Souza and colleagues predicted phenylalanine auxotrophy for many of the bacteria that grow in minimal media. Furthermore, the rate of "auxotrophies" was about the same for bacteria that are verified to grow in minimal media as for other bacteria. This suggests that most of their predicted auxotrophies are spurious.
