# Supplementary material for: Filling gaps in bacterial amino acid biosynthesis pathways with high-throughput genetics
Source: PLoS Genet. 2018 Jan 11;14(1):e1007147. doi: 10.1371/journal.pgen.1007147 (PMC5764234; doi:10.1371/journal.pgen.1007147)
Supplement: S2 Text — (PDF) [file pgen.1007147.s004.pdf]

S2 Text: Tests of growth without added amino acids or vitamins

To rule out growth due to the small amounts of nutrients that remain after transfer from rich media into minimal media, we performed multi-transfer growth experiments for seven bacteria (*B. phytofirmans*, *D. vulgaris* Miyazaki F, *H. seropedicae*, *M. adhaerens, P. inhibens*, *P. stutzeri,* and *S. meliloti*). After recovering each of these strains from the freezer in rich media, we transferred them into minimal media (with vitamins but no amino acids), grew them up, and transferred them 1-2 additional times. We observed robust growth of all strains after the final transfer into fresh minimal media. (For this test of *D. vulgaris*, we used 40 mM L-lactate and 20 mM sulfate rather than the usual 60 mM D,L-lactate and 30 mM sulfate.) Also, although we did not test *A. brasilense* Sp245 ourselves, it is reported to grow with nitrogen gas as its sole source of nitrogen [17], which implies that it can synthesize all 20 amino acids. We also tested growth of the seven bacteria in defined media with the vitamins omitted. Except for *S. meliloti*, they all grew without added vitamins. *S. meliloti* 1021 is reported to grow in minimal media (with no amino acids) if biotin and cobalt are provided [12]. The other six strains appear to be able to make all of the necessary vitamins. But it is difficult to be certain that none of our ingredients or glassware was contaminated with small amounts of vitamins. To test the possibility that our media was contaminated with low but sufficient concentrations of vitamins, we collected mutant fitness data for *B. phytofirmans, H. seropedicae, M. adhaerens, P. inhibens,* and *P. stutzeri* growing in defined media without any added vitamins. We identified auxotrophic phenotypes in more than one organism for mutants in the biosynthesis of NAD, thiamine, and biotin (for NAD biosynthesis genes, in *B. phytofirmans*, *P. inhibens*, and *P. stutzeri*; for thiamine biosynthesis genes, in *H. seropedicae* and *P. stutzeri*; and for biotin biosynthesis genes, in *B. phytofirmans, H. seropedicae, P. inhibens,* and *P. stutzeri).* This suggests that our media did not contain these vitamins at a concentration that was sufficient for efficient growth. Also, in *P. stutzeri*, which contains both a vitamin B12-dependent methionine synthase and a B12-independent methionine synthase, the B12-independent isozyme was important for fitness in the absence of vitamins (gene fitness under -3) but not in the presence of our vitamin mix, which included cyanocobalamin (gene fitness = -0.1). This suggests that our media did not contain sufficient vitamin B12. Our vitamin mixes also contained vitamin B6 (as pyridoxine), lipoic acid, riboflavin, folic acid, and pantothenate. For all five of these bacteria, the biosynthetic pathways for these vitamins appear to be essential for growth in rich media that contains yeast extract, which explains why we failed to identify auxotrophic mutants in these pathways. These bacteria may not be able to take up these vitamins.
