# Supplementary material for: Correction: Filling gaps in bacterial amino acid biosynthesis pathways with high-throughput genetics
Source: PLoS Genet. 2019 Apr 3;15(4):e1008106. doi: 10.1371/journal.pgen.1008106 (PMC6447180; doi:10.1371/journal.pgen.1008106)
Supplement: S3 Text — (PDF) [file pgen.1008106.s002.pdf]

S3 Text: Clear candidates whose mutants were not consistently auxotrophic

Most of the clear candidates (that is, genes that were identified by at least two out of three annotation resources as filling gaps in the IMG predictions) were either important for fitness in minimal media, or were likely essential in rich media, or the lack of an auxotrophic phenotype could be explained by genetic redundancy. Here we describe the two exceptions.

First, Psest_1986 from *P. stutzeri* contains a prephenate dehydrogenase domain (PF02153) fused to a 3-phosphoshikimate 1-carboxyvinyltransferase (TIGR01356). Although this gene is expected to be required for tyrosine biosynthesis, it was not important for fitness in most defined media conditions. The gene's mutant fitness was estimated from just two mutant strains, both of which lie in the linker region between the two domains. The location of these strains is striking because the linker region contains just 40 of the 747 amino acids of Psest_1986. Our interpretation is that both enzymatic activities are essential, and that these insertions were recovered because they allowed both domains to be expressed as separate proteins.

Second, DvMF_1902 from *D. vulgaris* contains a D-isomer specific 2-hydroxyacid dehydrogenase catalytic domain (PF00389) and is annotated as D-3-phosphoglycerate dehydrogenase by both KEGG and SEED. It is 39% identical to a characterized D-3-phosphoglycerate dehydrogenase (MMP1588; [59]). This activity is expected to be required for serine biosynthesis, as a related strain of *Desulfovibrio* appears to use the standard pathway of serine synthesis via phosphorylated intermediates 60]. Also, the isotope labeling of serine in another strain of *D. vulgaris* is consistent with the standard pathway [61]. Nevertheless, DvMF_1902 was not important for fitness in defined media (all fitness values were -0.5 or higher). Although we did not identify any other strong candidate genes for this activity, DvMF_1902 could be redundant with another dehydrogenase.

Additional References

59. Helgadóttir S, Rosas-Sandoval G, Söll D, Graham DE. Biosynthesis of phosphoserine in the Methanococcales. J Bacteriol. 2007 Jan;189(2):575–82.

60. Germano GJ, Anderson KE. Serine biosynthesis in Desulfovibrio desulfuricans. J Bacteriol. 1969 Sep;99(3):893–4.

61. Tang Y, Pingitore F, Mukhopadhyay A, Phan R, Hazen TC, Keasling JD. Pathway confirmation and flux analysis of central metabolic pathways in Desulfovibrio vulgaris hildenborough using gas chromatography-mass spectrometry and Fourier transform-ion cyclotron resonance mass spectrometry. J Bacteriol. 2007 Feb;189(3):940–9.
